# Geographic distribution of hospital beds throughout China: a county-level econometric analysis

**DOI:** 10.1186/s12939-016-0467-9

**Published:** 2016-11-08

**Authors:** Jay Pan, David Shallcross

**Affiliations:** 1 Health Economics, West China School of Public Health, Sichuan University, Chengdu, China; 2 Public Health in Developing Countries, London School of Hygiene and Tropical Medicine, Keppel Street, London, WC1E 7HT UK; 3 West China Research Center for Rural Health Development, Sichuan University, Chengdu, China

**Keywords:** Hospital bed capacity, Health equity, Health services geographic accessibility, Spatial analysis, China

## Abstract

**Background:**

Geographical distribution of healthcare resources is an important dimension of healthcare access. Little work has been published on healthcare resource allocation patterns in China, despite public equity concerns.

**Methods:**

Using national data from 2043 counties, this paper investigates the geographic distribution of hospital beds at the county level in China. We performed Gini coefficient analysis to measure inequalities and ordinary least squares regression with fixed provincial effects and additional spatial specifications to assess key determinants.

**Results:**

We found that provinces in west China have the least equitable resource distribution. We also found that the distribution of hospital beds is highly spatially clustered. Finally, we found that both county-level savings and government revenue show a strong positive relationship with county level hospital bed density.

**Conclusions:**

We argue for more widespread use of disaggregated, geographical data in health policy-making in China to support the rational allocation of healthcare resources, thus promoting efficiency and equity.

## Background

Healthcare access is recognized as a fundamental human right [1], and the geographic distribution of a healthcare delivery system is an important component of healthcare access [2]. Equitable geographic distribution of healthcare resources is believed to improve both health system quality [3–8], and economic efficiency [9]. Empirical work has shown that inequities in geographic distribution are associated with inequities in health outcomes.

Given the importance of both the absolute level and the relative distribution of healthcare resources, there is a growing literature aimed at understanding healthcare resource allocation patterns [5, 10–12]. In China, although authors have noted over-concentration of health resources in urban centres to the detriment of the rural population [13, 14], little empirical work has been published. Yet analysis of healthcare resource allocation would help to evaluate the impact of China’s health system reforms. For example, we know that recent health finance initiatives have transformed how healthcare is paid for: between 2001 and 2013, the share of public and social funds in healthcare financing increased from 40 to 66.1 %, bringing out-of-pocket spending close to levels observed in OECD countries [15]. A plausible hypothesis is that non-economic factors, including geographical barriers, have consequently become more important in determining healthcare access. However, the evidence that could test this and inform future policy is lacking.

Hence, the purpose of this study is, first, to describe inequities in the county level geographical distribution of hospital beds in China, and, second, to identify the determinants of hospital bed density at the county level in order to contribute to evidence-informed policy in China.

## Methods

### Data source

Our study used county level data on hospital beds and socio-economic characteristics. These data were obtained from the China County (City) Social and Economic Statistical Yearbook 2012, published by the Department of Rural Surveys of the National Bureau of Statistics of China. The China County (City) Social and Economic Statistical Yearbook reports data annually from the County (City) Social and Economic Statistical Reporting System [16], which covers all counties or county level cities from each of the 31 provinces in China, with the exception of Hong Kong SAR, Macao SAR and Taiwan. The reporting system collects data on a broad range of socio-economic indicators, which are compiled by local government departments.

This data source does not report on districts, which belong to the same administrative level as counties, but are predominantly found in urban centres. Hence, for our study, out of a total of 2853 county level administrative units in China in 2011, we obtained data on 2082 counties or county level cities. 39 counties (1.87 %) with missing data were later excluded from our analysis. Finally, the resulting sample consisted of 2043 counties from 31 provinces. The total sampled population was 990.4 million, or 73.5 % of the total population of China. Residents of districts, whose data were not available, account for the vast majority of those excluded.

### Variable(s) of interest

The key variable is the density of hospital beds, which is accepted as a proxy indicator for healthcare resources in the literature [5]. Hospital bed density is measured by the total number of beds in both hospitals and health centres at the end of the reporting period (Dec 31st 2011) per 1000 people registered as living in that county, based on their hukou. According to the statistical standard of the County (City) Social and Economic Statistical Reporting System, regular beds and care beds are included, but beds in outpatient observation rooms, pre-delivery beds, and beds for newborn babies in obstetric wards are excluded [16].

The major explanatory variables are residents’ savings per capita (Yuan), government revenue per capita (Yuan), percentage of urban population (%), and county land size (km^2^). Residents’ savings per capita is the total amount of savings in each county at the end of the year divided by the county population.

The state is the most important healthcare provider in China (90.3 % of all hospital beds were publicly owned in 2011 according to the National Bureau of Statistics of China [17], making county government revenue an important determinant of the availability of healthcare resources. Since county government revenue comprises both county level tax contributions and higher level (provincial and central) government transfers, it is not perfectly correlated with county economic development, and is thus included as an independent variable.

In China, there is an urban–rural dichotomy, with stark differences along a number of economic and social dimensions. To control for these differences, we included urban population share in our analysis. Finally, to control for differences in size among counties, we included county area as a geographical indicator.

### Measuring inequality in the distribution of hospital beds

We used the Gini coefficient to measure inequalities in the geographic distribution of hospital beds. This approach is widely used in the related literature [5, 10, 18–20]. The value of the Gini coefficient ranges between 0 and 1, with higher values indicating greater inequality [21].

### The determinants of hospital bed density across counties

#### Ordinary least squares (OLS) model

To examine the relationship between county characteristics and hospital bed density, we used a conventional OLS model. The regression equation takes the following form:

1 $$ {y}_i={X}_i^{\hbox{'}}\beta +{\varepsilon}_i $$

where, *i* indexes our counties, *y*_*i*_ is the number of hospital beds in county *i*, *X*_*i*_ is a vector of county characteristics, including savings per capita, government revenue per capita, percentage of urban population, and county area. The coefficients β measure the correlation between county features and hospital beds, and *ε*_*i*_ is the error term. Four possible functional forms (the log-log, linear, exponential, and semi-log) could interrelate the dependent and independent variables. In this study, a log-log functional form is assumed among variables, except for the percentage of urban population, where the relationship is assumed to be linear. We used the framework of Box-Cox transformation analysis to test this assumption [22], and the results indicated that our assumed form is preferred to other possible functional forms. In addition, significant heterogeneity may be present at the provincial level owing to different legal, institutional and cultural environments, and this could bias the estimation. Hence we included additional specifications with provincial fixed-effects.

#### Spatial models

Studies in other countries have revealed spatial autocorrelation of healthcare resources [3, 5, 10]. Since conventional regression analysis would not fully capture this characteristic, and that failure to do so could introduce significant bias into our conventional OLS model [23], we decided to test the spatial interdependence characteristics of hospital bed distribution in China. We used two commonly applied models proposed by Anselin - the spatial lag model and the spatial error model [23].

Based on Eq (1), the spatial lag model can be written as:

2 $$ {y}_i=\rho W{y}_i+{X}_i^{\hbox{'}}\beta +{\varepsilon}_i $$

where *ρ* is the spatial lag parameter, *W* is a *n × n* spatial weight matrix, and *Wy*_*i*_ is the hospital beds spatial lag variable. Thus, the estimation of the coefficient *ρ* reflects the slope of the reaction function, that is, how much county *i*’s hospital bed distribution is influenced by neighbouring counties.

The spatial error model can be expressed as:

3 $$ {y}_i={X}_i^{\hbox{'}}\beta +{\varepsilon}_i $$

with

4 $$ {\varepsilon}_i=\lambda W{\xi}_i+{\mu}_i $$

Eq (3) is the same as Eq (1), but is additionally combined with Eq (4), where *μ*_*i*_ is a spatially uncorrelated error term that satisfies the normal regression assumption, and $\xi$, is a term indicating the spatial component of the error term. The parameter *λ* indicates the extent to which the spatial component of the errors $\xi$ are correlated with one another for nearby counties, as given by the spatial weight matrix *W*.

We use the maximum likelihood approach for both models. If there is no spatial correlation, the parameter estimate (*ρ* or *λ*) will be statistically indistinguishable from 0, and the model will reduce to the standard OLS model where the individual observations are independent of one another, as in Eq (1). If there is a statistically positive value for the parameter (*ρ* or *λ*), it indicates that counties will tend to provide more hospital beds, if on average, their neighbours provide more, whereas a negative parameter value suggests that counties will tend to provide fewer beds, as their neighbours provide more.

If spatial interdependence does exist, it is preferable to determine which model provides the best fit using observed data rather than theoretical predictions. Thus, following the procedure proposed by Anselin [24], this paper proceeds by first estimating the β coefficients by OLS regression. Using the OLS residuals, we carried out three different tests, namely Moran’s I test, and two robust Lagrange Multiplier tests. The first tests for any spatial dependence, while the second and third test for spatial lag dependence and spatial error dependence respectively.

Statistical analyses were performed using GeoDa 1.4.6 [25], and Stata 12 [26], and maps were generated using ArcGis 10 [27].

## Results

### Sample description

Table 1 reports descriptive statistics for the sampled counties. The mean number of hospital beds per 1000 people was 2.849 in 2011, whereas the median value was 2.496, indicating positive skew. The highest hospital bed density was 70 times greater than the lowest, reflecting tremendous health resource inequalities across China.

**Table 1:** Descriptive Statistics of Data, China Counties, 2011

Variable	Mean	Standard deviation	Minimum	Median	Maximum
Hospital beds per 1000 people	2.849	1.710	0.362	2.496	26.605
Saving per capita (Yuan)	13981.950	13105.190	117.546	10712.200	177571.600
Government revenue per capita (Yuan)	1632.617	2467.760	96.552	879.000	39794.290
Percentage of urban population (%)	21.028	14.665	0.000	17.241	97.297
Area size (km^2^)	4205.038	9819.661	56.000	2075.000	202298.000
Sample size	2043				

### Geographic distribution

**Fig. 1:**
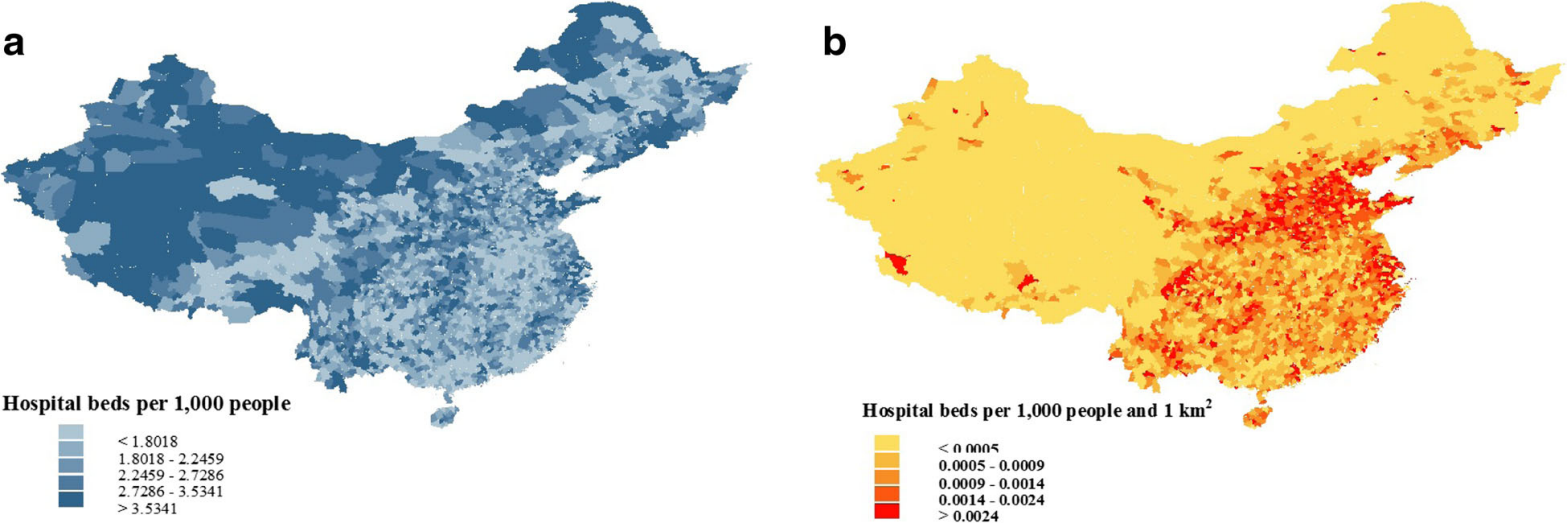
County Level Hospital Bed Density throughout China, 2011

Figure 1 contrasts two different measures of hospital bed density. In (a) counties are categorized into quintiles on the basis of hospital beds per 1000 people, with deeper shading representing higher bed density. By this measure, western counties had the highest bed densities. In (b) county level hospital bed densities are further divided by county area to reflect the fact that spatial distance to service provider is also a determinant of demand. Under this new measure, the former trend is reversed: now western counties have a much lower bed density compared with the more prosperous southeastern areas of the country.

By grouping the counties into five quintiles, we obscured any inequalities within the same quintile. In the following sections, we use Gini coefficients to provide a more detailed analysis.

### Inequalities by province

**Fig. 2:**
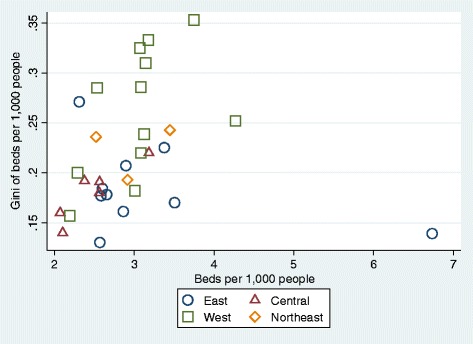
Relationship between Hospital Bed Density and Gini Coefficient by Province. Notes: Eastern provinces include Beijing, Fujian, Guangdong, Hainan, Hebei, Jiangsu, Shandong, Shanghai, Tianjin, and Zhejiang. Central provinces include Anhui, Henan, Hubei, Hunan, Jiangxi, and Shanxi. Western provinces include Chongqing, Gansu, Guangxi, Guizhou, Inner Mongolia, Ningxia, Qinghai, Shaanxi, Sichuan, Tibet, Xinjiang, and Yunnan. Northeastern Provinces include Heilongjiang, Jilin, Liaoning

Figure 2 reports the hospital bed density per 1000 people and the Gini coefficient for each province. Moreover, it uses the categorization of the China Statistics Bureau (which divides the country into four regions - east, central, west, and north-east - based on economic development and geographic location) [17] to investigate differences in inequality.

Figure 2 also reveals significant inter- and intra-regional inequalities in resource distribution. Western China is particularly disadvantaged - the six most inequitable provinces all belong to this region. However, geography alone does not fully explain this pattern, since some provinces in western China manage to distribute their resources more evenly, for example, Guangxi Province, which is actually the fourth best performer overall in terms of equity.

### Regression analysis

We utilized conventional OLS regression and spatial regression analysis to test whether spatial interdependence exists between counties, and to examine the relationship between county characteristics and hospital bed density.

Table 2 presents the regression estimates and the corresponding diagnostic tests. We first estimated the OLS regressions. Columns (1) and (2) show the results. All variables are in logs except urban population share which is in percentages. Even after including provincial fixed-effects, the coefficients on all four variables were statistically significant at the 1 % level.

**Table 2:** Regression Results for Hospital Bed Density, China Counties, 2011

	Log of hospital beds per 1000 people					
	OLS models		Spatial lag models		Spatial error models	
Variables	(1)	(2)	(3)	(4)	(5)	(6)
Log of saving per capita	0.175***	0.228***	0.144***	0.216***	0.233***	0.232***
	(10.693)	(12.985)	(8.852)	(12.479)	(13.321)	(13.321)
Log of government revenue per capita	0.107***	0.119***	0.096***	0.110***	0.107***	0.105***
	(8.593)	(9.216)	(8.083)	(8.647)	(8.622)	(8.254)
Percentage of urban population (%)	−0.003***	−0.003***	−0.003***	−0.002***	−0.003***	−0.002***
	(−4.180)	(−3.073)	(−3.752)	(−2.905)	(−4.080)	(−2.154)
Log of area size (km^2^)	0.082***	0.034***	0.030***	0.013	−0.009	−0.006
	(8.332)	(2.981)	(3.167)	(1.163)	(−0.788)	(−0.477)
Rho (ρ) / Lambda (λ)			0.724***	0.486***	0.905***	0.925***
			(24.329)	(8.751)	(33.334)	(40.683)
Provinces dummies	No	Yes	No	Yes	No	Yes
Sample size	2043	2043	2043	2043	2043	2043
R^2^	0.253	0.394	0.329	0.411	0.403	0.439
Log likelihood	−936.862	−723.352	−839.045	−698.927	−731.663	−669.284
Akaike info criterion	1883.72	1516.7	1690.09	1469.85	1473.33	1408.57
*Spatial model diagnostic tests*						
Moran’s I (error)	43.144***	19.880***				
Robust Lagrange Multiplier (lag)	43.214***	0.424				
Robust Lagrange Multiplier (error)	1378.343***	115.737***				

(1) ***,** and * denote 1, 5 and 10 % significance levels, respectively; (2) Since there are 31 provinces in our dataset, 30 provinces dummies are added to the corresponding regressions; (3) All spatial model diagnostic tests are based on OLS

We performed Moran’s I test to test for spatial dependence. The test results are listed at the bottom of Table 2. Moran’s I statistic was highly significant despite the provincial dummies, indicating that spatial autocorrelation was large enough to create misspecification problems with the conventional OLS method. However, Moran’s I statistic does not identify which model should be used. For this, we used two robust Lagrange multiplier tests. The LM-Lag and LM-Error statistics were both highly significant when there was no controlling for provincial-level effects, however, with provincial dummies only the LM-Error statistic was significant. This indicates that the spatial error model is preferred.

An alternative way to choose the correct spatial model is to inspect three regular statistics: R-squared, log likelihood ratio, and Akaike info criterion (AIC). These statistics are reported in Columns (3) – (6) following the corresponding regression models. The R-squared for the spatial error model is larger than for the spatial lag model, suggesting it can explain more of the variation in hospital beds. The log likelihood ratio and AIC are respectively larger and smaller compared with the spatial lag model, both of which corroborate our finding that the spatial error model fits best. Since the robust LM test and the regular statistics concur that the spatial error model is superior, the following discussions are based on the spatial error model estimates. Specifically, we focus on Column (6), for which provincial dummies were included.

The spatial autoregressive coefficient (*λ*) of the spatial error model is highly significant (*p* < 0.0001). Indeed, even after controlling for county characteristics such as local economic development, the spatial correlation estimate is 0.925. This value is remarkably high and shows the extent to which hospital beds are clustered at the county level in China.

The estimated coefficients for savings per capita and government revenue per capita are positive and statistically different from zero at the 1 % significance level. The elasticities of resident savings and government revenue are 0.232 and 0.105 respectively. That is to say that a 10 % increase in savings is associated with a 2.32 % increase in the average hospital bed density, while a 10 % increase in government revenue is associated with a 1.05 % increase in hospital bed density. Although government revenue comprises county level tax income, and is thus correlated with economic development, it also comprises transfers from central government, hence the government may still be able to increase hospital bed availability even when local economic conditions are unfavorable. Our finding of an independent effect is consistent with previous work highlighting the role played by higher level fiscal policy in the development of local health infrastructure [28].

The coefficient for urbanization reaches statistical significance, whereas that for county size does not. The negative value of the urbanization coefficient suggests that hospital bed density decreases as urbanization increases. However, the effect size is negligible – according to the model, even if a county transformed from completely rural to completely urban, the hospital bed density would only decrease by an average of 0.2 %. Hence, once economic factors and spatial interdependence have been excluded, urbanization appears not to play a role in determining hospital bed density.

## Discussion

This paper employed county-level data from 31 provinces in China in 2011 to describe inequalities in hospital bed density, and to explore county-level determinants. We mapped national patterns of hospital bed density at the county level, and used Gini coefficients to examine within-province disparities. OLS and spatial models were applied to identify the determinants of hospital bed density.

Our paper has three major findings. First, despite ongoing healthcare reforms, substantial inequities in the geographical distribution of healthcare resources remain. Gini coefficient analysis suggests that provinces in western China are particularly disadvantaged in this regard. This finding is consistent with previous studies that have looked at regional patterns of healthcare resource distribution [29, 30]. As a key healthcare resource, hospital beds not only inform us about supply-side inputs into the health system, but can also predict demand-side outcomes such as healthcare access. Hence, the uneven geographic distribution that we have described is likely to create differential utilization of healthcare services, leading to inequalities in health outcomes. Furthermore, if hospital bed density is calculated on the basis of both county population and area – a commonsensical approach – western China has lower average resource levels than other regions. This fits with broader patterns of socio-economic deprivation in western China that have been noted elsewhere [31]. Thus, owing to the sparseness of its population, in order to achieve levels of spatial accessibility comparable to those in central and eastern China, western China may actually need a greater number of hospital beds per unit population. This serves as a reminder that when measuring health resource density, policy makers should consider the merits and interpretations of different definitions.

Second, geographical clustering is an important feature of hospital bed distribution in China, and the direction of the clustering is such as to exacerbate any existing resource inequalities. If we assume that patients can access healthcare facilities in neighbouring counties, the clustered nature of hospital bed distribution points to a “Matthew effect (accumulated advantage)” in healthcare resource allocation. In other words, not only do residents of counties with high hospital bed densities have good access to healthcare in their own county, they are also more likely to be near counties with plentiful healthcare resources. The opposite situation holds for residents of low-density counties. To our knowledge, this is a novel finding in the Chinese context. However, it is highly plausible given China’s health finance policies, which place a considerable burden of income-generation on individual healthcare facilities [28]. Without market intervention, this will tend to produce a scenario where hospitals in wealthier regions can afford to invest in healthcare resources, whereas those in poorer regions cannot.

Third, both local economic development and public sector investment are associated with hospital bed density, albeit with different strength. This finding reflects structural features of China’s mixed public-private healthcare financing arrangements. In particular, the fact that local economic development was the more powerful factor in our model - a 10 % increase in local savings per capita yielded a 2.32 % increase in hospital bed density - arguably points to the ongoing influence of out-of-pocket spending, especially in hospitals, which belong to higher tiers of the healthcare system, and thus have correspondingly higher expenses that are only partially covered by typical insurance plans [32]. Nevertheless, local government revenue, which partially derives from central government transfers [28], was an independent factor in our model, and thus also plays an important role in resource allocation. Since the launch of the latest round of healthcare reform in 2009, the Chinese government has increased investment in public hospitals, while encouraging the establishment of private hospitals. Although private investment has undoubtedly led to an overall increase in health resource funding, it has done little to address geographical disparities. Limited government resources would therefore be most effectively employed in disadvantaged counties, for example, through the intensification of special-purpose grants from the central government to economically under-developed areas. The government could also consider introducing policies to attract investment to such regions.

Our study should be considered in light of its limitations. One important limitation is that the data only covered 73.5 % of the national population owing to the unavailability of data on districts. This means that large urban centres are under-represented in the analysis, and that the provincial averages of hospital-bed density and within-province inequalities presented here are thus likely to be underestimates. On the other hand, we do not anticipate that the inclusion of data from districts would seriously undermine any of our key findings. The second limitation is that our analysis only considers hospital bed distribution, and thus neglects outpatient health and public health. However, as noted above, the available evidence suggests that the distribution of public and primary healthcare resources in China differs little from what we found for hospital beds, and thus fits into a larger pattern of geographical inequalities [31, 33]. The third limitation is that our definition of county population (and thus the denominator in our hospital bed density calculations) was based on hukou registration, thereby ignoring any effects due to regional migration. Considering that the overall direction of population movement in China in recent years has been from rural to urban centres, this simplification is likely to have resulted in the under-estimation of hospital bed density for the primarily rural counties included in the sample. Moreover, this effect is likely to be even stronger for western China, since western China has been a region of net outward migration to more developed areas in central and eastern China. Nevertheless, we consider that the size of the regional disparity in our study is large enough that migration effects alone could not entirely account for it. Finally, we note that our analysis is cross-sectional, and thus unable to report trends in the evolution of hospital bed density over the course of China’s recent healthcare reforms. This is a significant drawback, since it prevents us from drawing conclusions about whether the reforms have been effective in reducing disparities.

In conclusion, our study identifies geographical inequalities in healthcare resources that remain to be addressed. Of particular concern is the high degree of spatial clustering, which threatens achievement of equity. Although, broadly speaking, both economic development and government revenue have a positive role to play in improving the health system, options may be constrained at the local level. In areas of economic deprivation, it is doubtful whether economic development is a feasible or sufficiently timely solution to ensure equitable healthcare access, and the government may need to intervene more strongly to overcome entrenched disadvantage.

## Conclusions

Based on our analysis, we make the following recommendations. First, disaggregated county-level data permits more sophisticated analysis of access to healthcare, and should be used as part of comprehensive monitoring and evaluation of government healthcare policy. Second, at the provincial and county level, the government should carefully consider what mix of funding levels, sources and mechanisms can best address inequities in health resource distribution. In particular, the government should pay attention to the negative impact of current resource clustering patterns, and seek to mitigate this problem through appropriate use of private investment and direct public sector investment, including structural adjustments to local health financing policies where necessary.
